# The Characterisation of Pax3 Expressant Cells in Adult Peripheral Nerve

**DOI:** 10.1371/journal.pone.0059184

**Published:** 2013-03-19

**Authors:** Judith A. Blake, Melanie R. Ziman

**Affiliations:** 1 School of Medical Science, Edith Cowan University, Joondalup, Western Australia, Australia; 2 School of Pathology and Laboratory Medicine, University of Western Australia, Nedlands, Western Australia, Australia; University of Edinburgh, United Kingdom

## Abstract

Pax3 has numerous integral functions in embryonic tissue morphogenesis and knowledge of its complex function in cells of adult tissue continues to unfold. Across a variety of adult tissue lineages, the role of Pax3 is principally linked to maintenance of the tissue’s resident stem/progenitor cell population. In adult peripheral nerves, Pax3 is reported to be expressed in nonmyelinating Schwann cells, however, little is known about the purpose of this expression. Based on the evidence of the role of Pax3 in other adult tissue stem and progenitor cells, it was hypothesised that the cells in adult peripheral nerve that express Pax3 may be peripheral glioblasts. Here, methods have been developed for identification and visualisation of Pax3 expressant cells in normal 60 day old mouse peripheral nerve that allowed morphological and phenotypic distinctions to be made between Pax3 expressing cells and other nonmyelinating Schwann cells. The distinctions described provide compelling support for a resident glioblast population in adult mouse peripheral nerve.

## Introduction

The *paired-box homeotic gene 3* (*PAX3*) encodes the PAX3 transcription factor that is known to regulate transcriptional activation or repression of a broad spectrum of downstream genes in a variety of developmental pathways. *PAX3* functions after embryogenesis relate to regulatory roles in the ontogeny of stem cells throughout the postnatal lifespan of the organism. The roles of *PAX3/Pax3* are well defined across a variety of adult tissue lineages [1]–[12]. From these studies, it can be concluded that the overarching purpose for continued expression of *PAX3/Pax3* in adult tissues is primarily for maintenance of a progenitor cell population. In adult progenitor cells it is said that *PAX3/Pax3* protects the ‘stemness’ of the cell through regulation of downstream target genes involved in the maintenance of an undifferentiated phenotype and in its absence, cells acquire the characteristics of a mature cell [13].

*Pax3* is known to be expressed in a characteristic, temporal pattern in Schwann cells of the developing peripheral nervous system [14]. Kioussi and colleagues [14] report that *Pax3* RNA is associated with nonmyelinating Schwann cells (NMSCs) of 30 day old mouse sciatic nerve; this report of continued *Pax3* expression in adult cells of neural crest origin was thought intriguing. Therefore, investigations focused on determination of the *Pax3* mRNA transcripts and double-labeling of Pax3 with other early immature Schwann cell markers in normal 60 day old mouse sciatic nerve and results demonstrate that cells that express Pax3 are characterised by a peripheral glioblast phenotype.

## Results

### *Pax3* mRNA Transcripts are Expressed in 60 Day Old Mouse Sciatic Nerve

There are conflicting reports about the expression of *PAX3/Pax3* in Schwann cells of adult peripheral nerve [14]–[16]; so, the initial aim of the investigations was to report the *Pax3* mRNA transcripts in normal mouse sciatic nerve. To identify all possible *Mus musculus* mRNA transcripts, the mouse genome sequence available on the NCBI was interrogated for all possible splice sites. Three mouse transcripts have been sequenced to date; *Pax3c* and *Pax3d* are expressed in embryonic cells of the myogenic and melanogenic lineages [17] and *Pax3?8*, which encodes a transcriptionally inactive isoform, is expressed in embryonic myogenic precursors [18]. Barber et al. [17] have reported a *Pax3f* transcript, expressed in the embryonic day 9.5 mice and although exact sequence data is unavailable, it is thought that the transcript is generated by splicing exon 5 directly to exon 9 using the known splice donor and acceptor sequences.

To delineate whether the production of additional mouse transcripts of *Pax3* is possible, a comparison of human and mouse nucleotide sequences was undertaken using the NCBI BLAST database (http://blast.ncbi.nlm.nih.gov/Blast.cgi) to search for mouse consensus donor and acceptor splice sequences contained within the *Pax3* locus. The amino acid sequences from 197–215 of human PAX3a or 197–206 of PAX3b are not homologous to those of mouse Pax3 [19], [20] and there is no record of a *Mus musculus* transcript *Pax3a* or *Pax3b*. The mouse *Pax3* gene shows a lack of consensus splice site elements required for production of homologous *Pax3e, Pax3g* and *Pax3h* transcripts as those produced in humans; moreover, the mouse *Pax3* genomic sequence diverges from the human gene in the 3′ region from which these transcripts are produced and shows less than 70% homology to the human sequence (Murine clone RP24-529B23 Chromosome 1).

**Figure 1:**
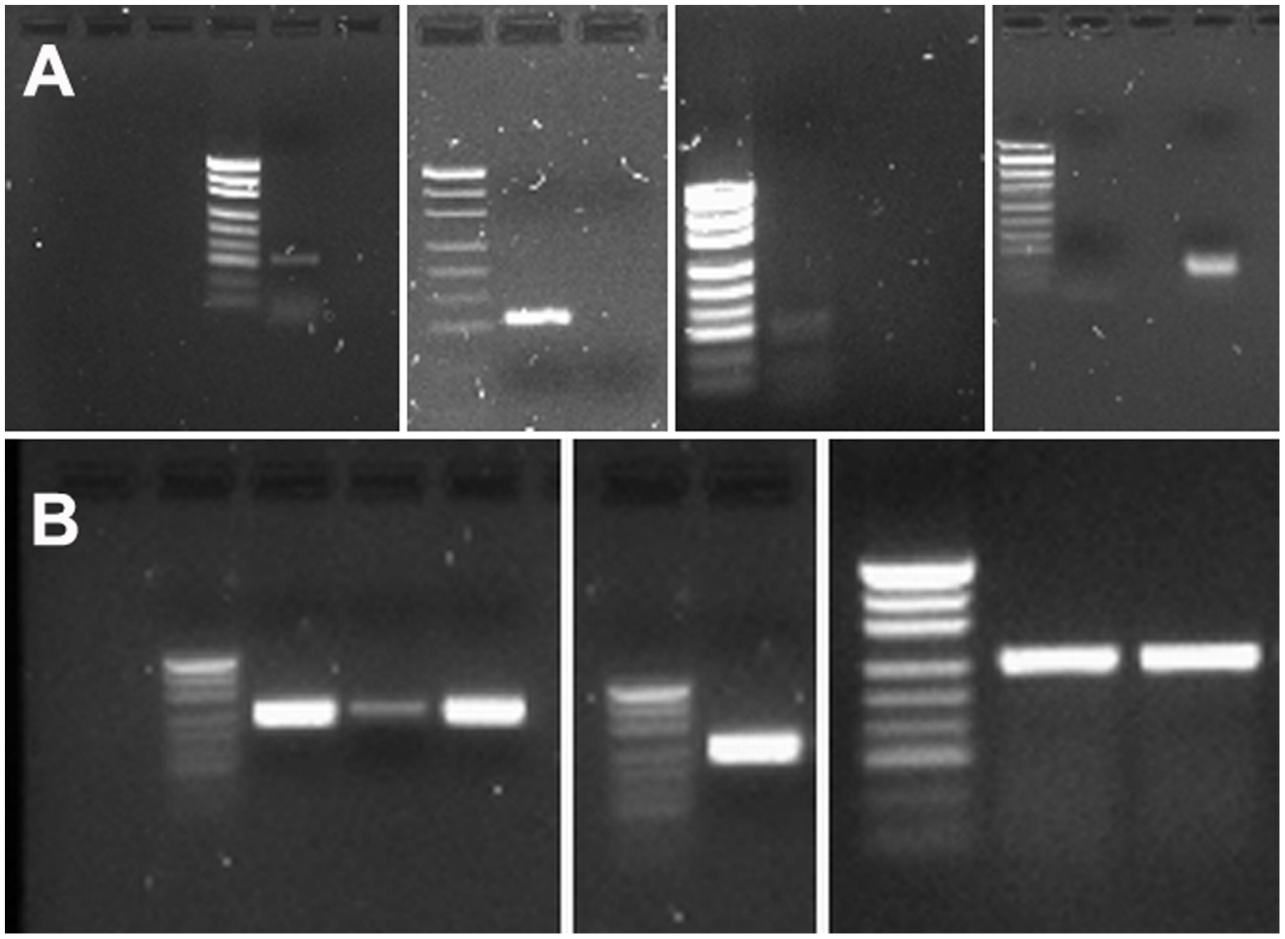
*Pax3* transcripts are expressed in normal adult mouse peripheral nerve. Gel electrophoresis of PCR amplification products of *Pax3* transcripts from normal mouse sciatic nerves. All PCR products are compared to pUC DNA ladder. **A)** *Pax3c* (117 bp) was expressed in 3/6 nerves tested while *Pax3d* (97 bp) was expressed in 1/6 nerves tested. *Pax3* products were not amplified in 2/6 nerves tested. **B)** Positive controls for relative amounts of *Gapdh* product amplified from the total RNA of the six nerve lysates are shown.

As such, specific primers were designed to amplify the mRNA of mouse *Pax3c*, *Pax3d*, *Pax3f* and *Pax3?8* transcripts. RT-PCR results confirmed that 2 alternate *Pax3* mRNA transcripts were expressed in 60 day old mouse sciatic nerve (n = 6). *Pax3c* or *Pax3d* transcripts were detectable in 4/6 individual nerves, although co-expression of both transcripts was not observed. In 2/6 nerves analysed, *Pax3* mRNA was undetectable. In all nerves tested, PCR amplification of *Pax3?8* and *Pax3f* mRNA products were undetectable (Fig. 1).

### The Morphology of 60 Day Old Mouse NMSCs of Sciatic Nerve

In the adult peripheral nervous system, C-fibre neurons are not myelinated and are organised into a bundle in which many nerve fibres are ensheathed by one NMSC for conduction of peripheral afferent signals. NMSCs have a characteristic morphology that consists of long branching networks of cytoplasmic processes which coalesce in a plexiform manner with adjacent nonmyelinated bundles [21], [22]. To date, the morphology of mouse NMSCs that make up nonmyelinated bundles have been loosely characterised.

In this study, mouse NMSCs were clearly visualised by fluorescence microscopy of teased sciatic nerve fibre specimens (data not shown); the morphology of the cell is stated as 2–4 µm in diameter across the cytoplasmic extensions and 4–5 µm in diameter across the nuclear region. The length of the cell is between 80–200 um and the nucleus is between 12–20 um in length. The nucleus of the NMSC is centrally located (as opposed to the peripheral location of the nucleus of a myelinating Schwann cell) and the nonmyelinated C-fibres that traverse longitudinally across the NMSC nucleus form it into a characteristic ‘cigar’ or spindle shape such as has been described for rat NMSCs [23].

**Figure 2:**
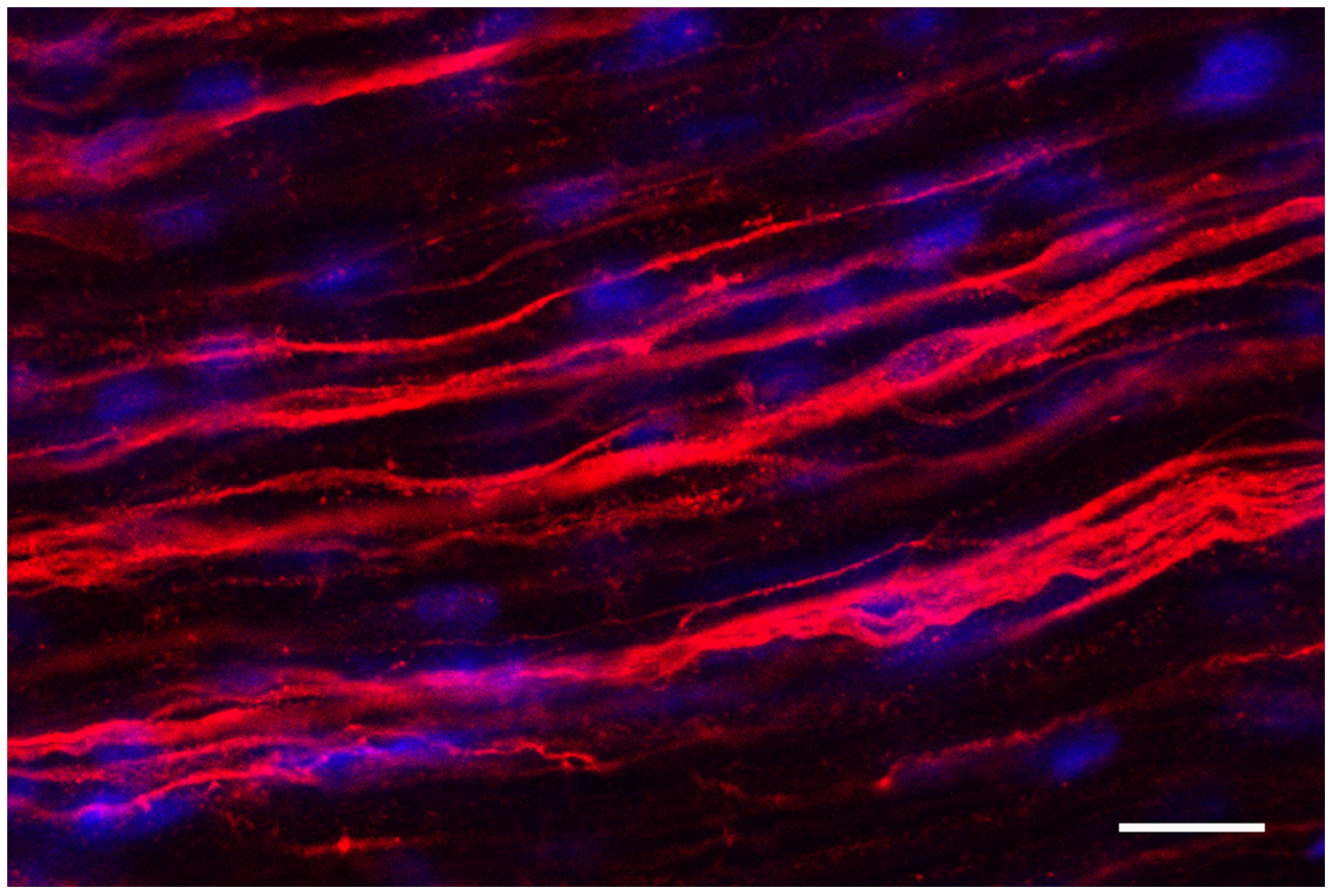
The complex structure of nonmyelinated nerve bundles in normal adult mouse peripheral nerve. A micrograph of a whole mount sciatic nerve fascicle preparation labeled with p75Ngfr (red) reveals the plexiform comingling and exchange of NGF-dependent C-fibres between adjacent nonmyelinated bundles. Cell nuclei are visualised using Hoechst DNA dye (blue). Images were acquired using scanning laser confocal microscopy. *Optical plane = 1.5 µm. Pinhole aperture = 3.0. Scale bar represents 20* *µm.*

It is known that nonmyelinated bundles contain two classes of C-fibres, those dependent on nerve growth factor (NGF) that express low-affinity nerve growth factor receptor (p75Ngfr) and those dependent on glial-derived neurotrophic factor that express glial-derived neurotrophic factor family receptor-α1 [22]. It is also known that p75Ngfr is expressed on the NMSC plasmalemma adjacent to a NGF-dependent C-fibre it ensheathes [23]; therefore, p75Ngfr was chosen to label NMSCs associated with NGF-dependent C-fibres. Here, the plexiform nature of nonmyelinated NGF-dependent C-fibres, described by Carlsen, Behse [21] in human and Murinson et al. [22] in rat, was seen in 60 day old mouse by the immunofluorescence labeling (Fig. 2).

### Characterisation of Cells that Express Pax3 in Normal Adult Mouse Sciatic Nerve

RT-PCR results verified that *Pax3c* and *Pax3d* transcripts were present in 60 day old mouse sciatic nerve (Fig. 1), thus, it remained to confirm the presence of the proteins encoded by these transcripts in the mouse tissue. A mouse monoclonal IgG2a isotype-specific antibody directed at amino acids that form the transactivation domain of the Pax3 protein [24] was employed and when used with the isotype-specific anti-mouse IgG2a secondary antibody, non-specific background staining of endogenous mouse tissue IgGs and other components was minimised.

**Figure 3:**
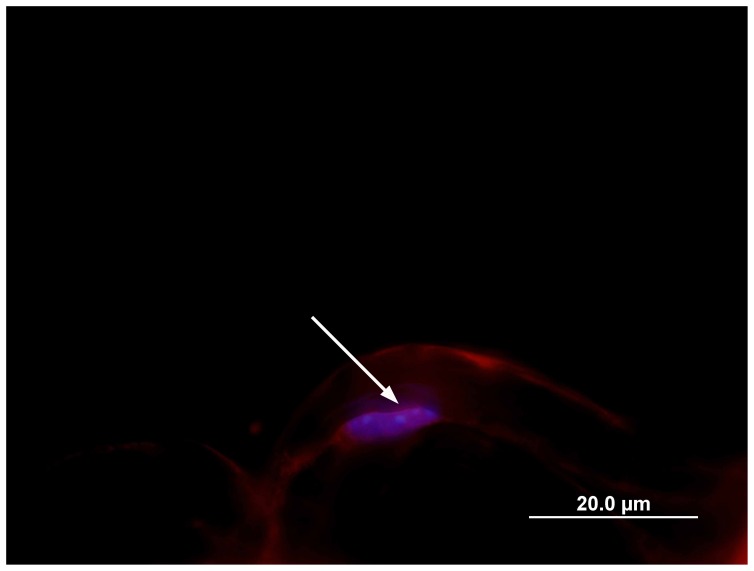
Krox24 immunofluorescence in adult mouse peripheral nerve. A sciatic nerve fascicle post-fixed for 2 hours in PFA was labeled with an antibody targeted at the Krox24 transcription factor. In this micrograph, a myelinating Schwann cell nucleus (indicated by the arrow) shows Krox24 positivity.

Development of the Pax3 immunofluorescent labeling method commenced using frozen sections of mouse sciatic nerve pre- and/or postfixed with 4% w/v paraformaldehyde (PFA) and a secondary indirect immunofluorescence procedure. In both tangental and longitudinal sections, a nuclear Pax3 label was undetectable. As indicated by the RT-PCR results, Pax3 expression levels were expected to be relatively low, thus, a tertiary (avidin/biotin) indirect immunofluorescence procedure was also performed on the frozen sections. When this method was analysed, levels of non-specific background staining were high and a nuclear Pax3 label remained undetectable (data not shown). Next, individual 2 mm lengths of fascicles from adult mouse sciatic nerve were pre- and/or postfixed in PFA and teased into individual Schwann cell/axons and indirect immunofluorescence methods were tested for Pax3 labeling. Various tissue permeabilisation protocols were also assessed as to their effects on cellular and extracellular integrity, nonspecific staining and intensity of immunofluorescent Pax3 label. Results showed that all methods trialed had a Pax3 label of low intensity and, in many specimens, Schwann cell structure was not optimal (data not shown). Positive control samples were processed during each of the teased fibre immunolabeling experiments using Krox24, a transcription factor reported expressed in Schwann cells of adult peripheral nerve [25], [26]. In the positive control samples, nuclei that expressed Krox24 were clearly distinguishable and strongly immunofluorescent (Fig. 3). It was thus concluded that the PFA fixation method was linked to difficulties associated with the lack of optimal Pax3 immunofluorescent labeling in both the frozen and teased fibre samples prepared.

**Figure 4:**
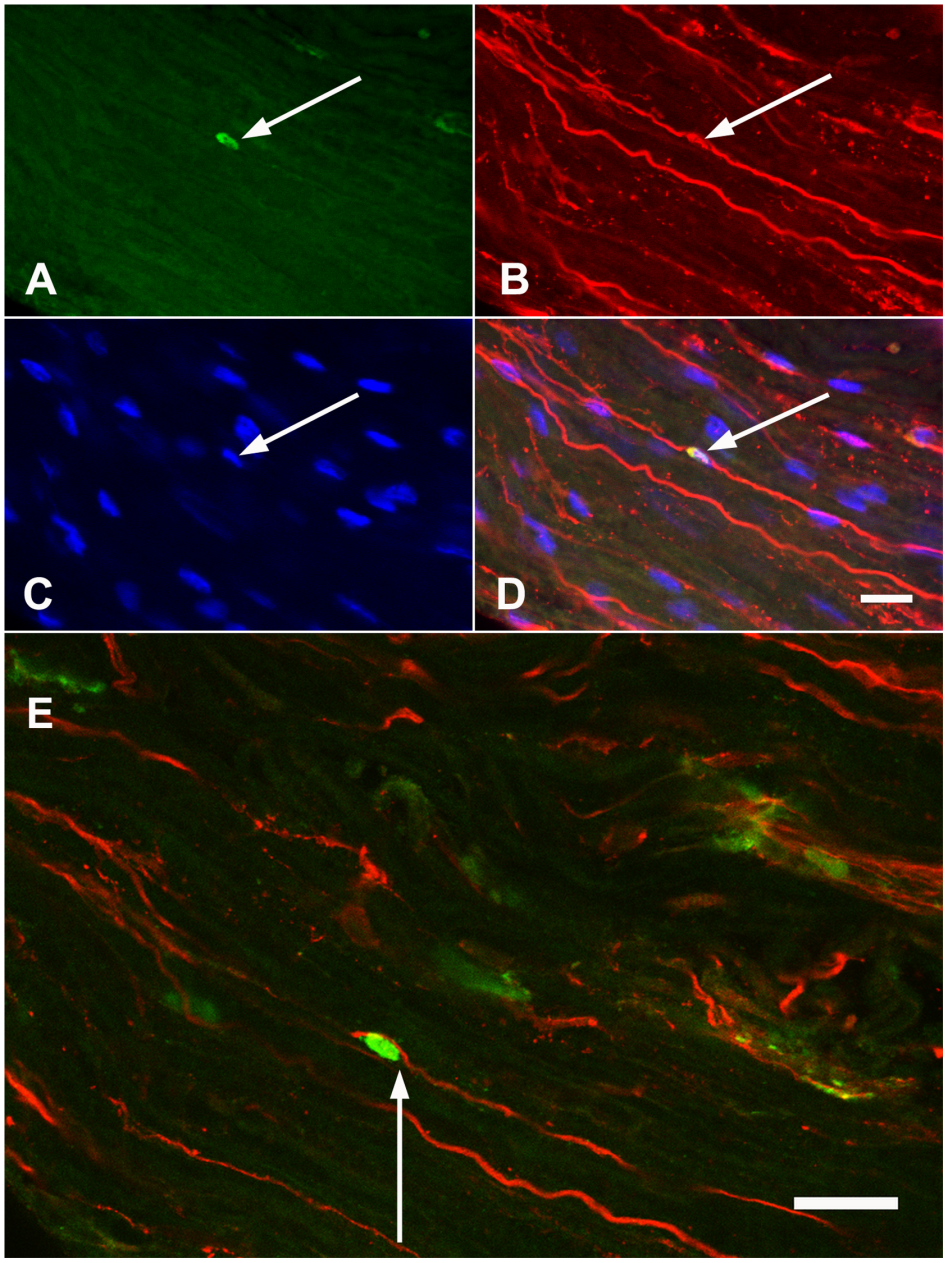
p75Ngfr and Pax3 co-localisation in normal adult mouse peripheral nerve. **A)** The Pax3 expressing cell (indicated by the arrow) is oval and has nuclear expression of p75Ngfr **(B)**. **D)** The merged images of Pax3 (**A**), p75Ngfr (**B**) and Hoechst DNA dye (**C**) immunofluorescence. **E)** The Pax3+ nucleus indicated (green) was imaged on a different focal plane than **A-D** such that it could be more clearly evident that it does not have p75Ngfr cytoplasmic extensions (arrow). Images were acquired using scanning laser confocal microscopy. *Optical plane = 1.5 µm. Pinhole aperture = 3.5. Scale bar represents 20* *µm.*

**Figure 5:**
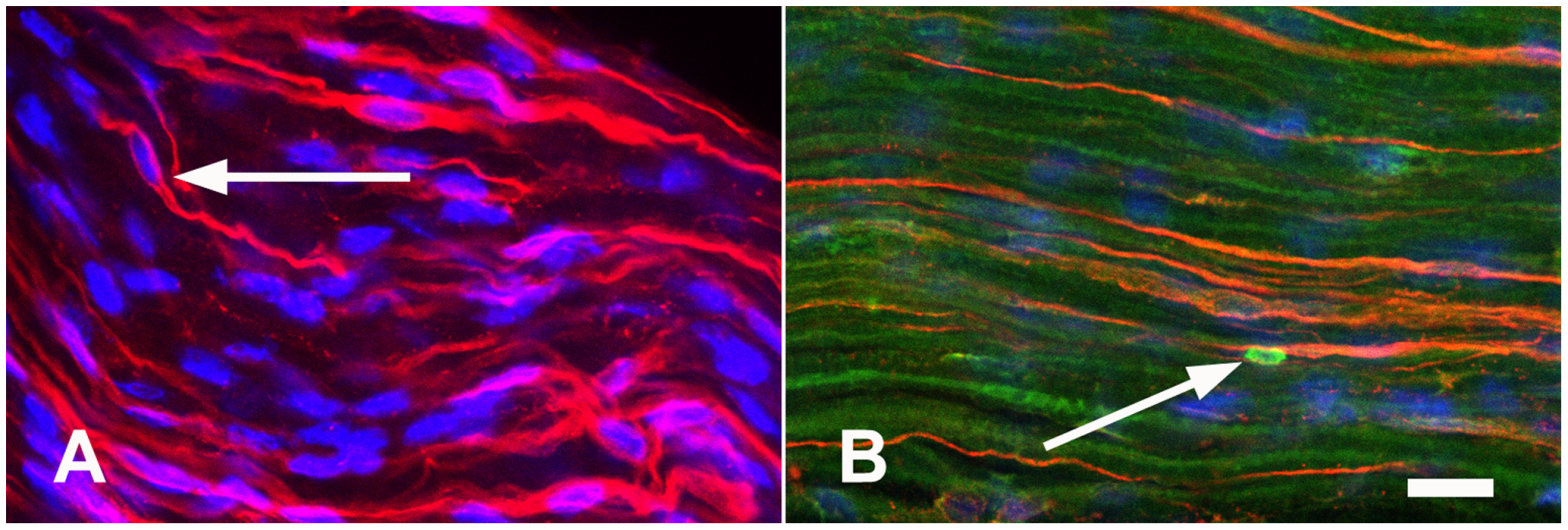
Distinctions between Pax3 expressant cells and the NMSC associated with NGF-dependent C-fibres. A) The cell indicated by the arrow has the characteristic morphology of the NMSCs associated with NGF-dependent C-fibres. Note the spindle shaped nucleus (blue) and p75+ cytoplasmic extensions (red). **B)** The cell indicated by the arrow typifies the morphology of the Pax3+ cells imaged in the study. The nucleus is oval and there are no associated p75+cytoplasmic extensions apparent.

An alternate method, consisting of a short post-fixation of dried whole mount sciatic nerve fascicles with 4°C acetone was subsequently found to preserve both tissue morphology and Pax3 antigenicity, therefore, a Pax3 labeling procedure was developed using this method of fixation and analysed using scanning laser confocal microscopy. Results showed strong Pax3 immunoreactivity in cell nuclei randomly distributed along the length of the 60 day old sciatic nerve trunk. In the whole mount mouse sciatic nerve fascicle specimens analysed, relatively 2% of cell nuclei were positive for Pax3 when compared to the total number of Hoechst stained nuclei visible along the length of the nerve (Fig. 4D, 5B); in the nerve fascicle preparations examined (approximately 2 mm length) there was an average of 9 cells per specimen that showed Pax3 positivity. Notably, the Pax3 expressing cells did not have a characteristic NMSC morphology. The shape of the nucleus was distinctly oval or round and co-localisation with p75Ngfr revealed that Pax3+ cells lacked p75Ngfr labeled bipolar cytoplasmic extensions (Fig. 5). In the three nerve preparations examined for colocalisation of Pax3 and p75Ngfr, 100% of the Pax3+ cells (n = 28) co-expressed p75Ngfr in the nucleus (Fig. 4B). It was evident that relatively 98% of Schwann cells associated with NGF-dependent C-fibres did not express Pax3.

**Figure 6:**
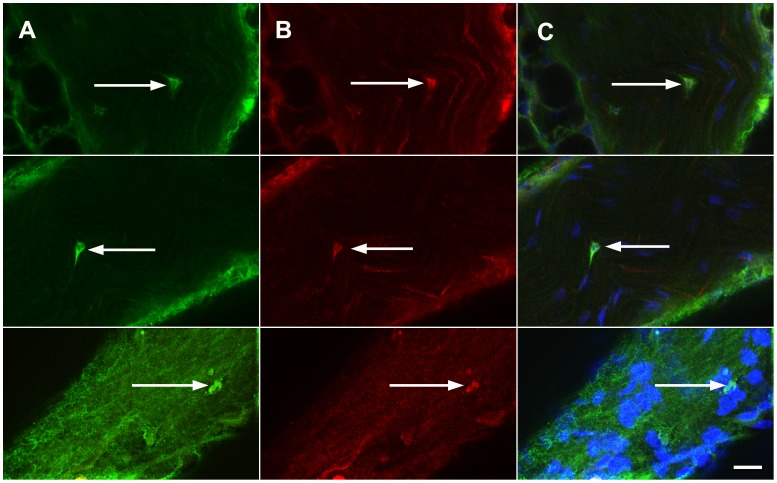
Transcription factors Pax3 and Sox2 co-localise in the nuclei of cells of adult mouse peripheral nerve. Whole mount sciatic nerve fascicles co-immunolabeled with Pax3 (green), Sox2 (red) and Hoechst DNA dye (blue) revealed that Pax3 expressant nuclei co-express stem cell marker Sox2 (indicated by the arrows). Images were acquired using scanning laser confocal microscopy. *Optical Plane = 1.5* *µm. Pinhole aperture = 3.5. Scale bar represents 20* *µm.*

It was hypothesised that cells of adult nerve that express Pax3 would co-express peripheral glioblast markers, therefore co-localisation studies were performed using antibodies against Pax3 and a marker of neural crest cells, transcription factor SRY-related high-mobility group box-2 (Sox2). To date, Sox2 expression has been thought limited to embryonic glioblasts [27]; however, it is detected here for the first time in mouse sciatic nerve of 60 day old animals. Across the three Pax3 and Sox2 double-labeled whole mount sciatic nerve fascicles examined, 100% of cells that had nuclear Pax3 expression also had nuclear Sox2 expression (n = 22) (Fig. 6) and again, cells that had Pax3/Sox2 positivity were approximately 2% of the total cells imaged.

## Discussion

This study was primarily concerned with the expression of a developmental transcription factor, Pax3, in adult mouse peripheral nerve. No studies have discussed Pax3 protein expression in normal adult mouse NMSCs and thus it was compelling to investigate and contemplate the implications of *Pax3* expression, as previously reported, knowing the roles of *Pax3*/Pax3 across other adult lineages such as melanocyte and skeletal muscle. It is important to note that initial development of the methods were based on an assumption that all of the P60 NMSC of peripheral nerve would express *Pax3*/Pax3, as it had been reported previously that the expression in P30 nerves was linked to inhibition of key myelination genes [14]. Our findings, which refute this, were tested using a variety of rigorously optimised methods. Taken together, the immunofluorescent labeling and RT-PCR results support one of the key findings of the study, namely, that Pax3 expression is demonstrated in only 2% of adult NMSCs. That Pax3 does not have a continuing role in the inhibition of myelination genes within adult NMSCs after P30 is supported by reports that cell fate specification to the myelinating phenotype occurs between E16 and birth and is principally linked to associated nerve fibre caliber and dose-dependent secretion of axonal factors [28]–[30].

The Pax3 expression pattern seen in adult peripheral nerve is similar to that seen in adult skeletal muscle where Pax3/7 expressing stem (satellite) cells account for up to 4% of the total myonuclei [31]. That *Pax3* transcripts were below the level of detection in some of the nerves tested here by RT-PCR, was confirmed by the fact that approximately 2% of cells within the nerve trunk express Pax3 at age 60 days. These findings are in agreement with other studies which failed to label *Pax3/*Pax3 in adult peripheral nerve [15], [16].

### Pax3 Expressing Cells in Adult Mouse Peripheral Nerve Co-express Stem Cell Markers

Based on the evidence of the role of Pax3 in other adult tissue stem and progenitor cells, and taken together with evidence that a population of cells exist in adult peripheral nerve that express Pax3 [14], it was hypothesised that these Pax3+ cells may be peripheral glioblasts. Therefore, aims of the investigations continued to focused on identification, visualisation and initial characterisation of the cells of adult mouse peripheral nerve that express Pax3. The most significant finding is a population of cells that co-express Pax3, Sox2 and p75Ngfr have been identified. These proteins are commonly expressed in multipotent cells in a variety of tissues [32]–[35] and while the role of Pax3 in Schwann cells remains largely undetermined, its overarching role in other tissues is maintenance of progenitor cells across the life span [9]. In Schwann cells, *Sox2* has been shown to increase responsiveness to proliferative stimuli, prevent myelin gene expression and inhibit differentiation [27], [36]. *SOX2* is one of the four Yamanaka factors, or genes whose expression is artificially forced to induce non-pluripotent adult somatic cells into pluripotent stem cells (iPSCs) *in vitro.* In the progress toward clinical application of iPSCs, both *SOX2* and *PAX3* have key roles in the generation, identification and maintenance of patient-specific iPSCs *in vitro* [37]–[39]. The demonstration of the novel subset of cells described in these investigations, although preliminary, is thought initial, sound evidence of the existence of peripheral glioblasts in adult mouse peripheral nerve.

### Pax3 Expressing Cells are Distinct from Other NMSCs of Adult Peripheral Nerve

Labeling procedures allowed several distinctions to be made between Pax3 expressing cells and other NMSCs. Firstly, it was seen that the Pax3+ cells co-expressed p75Ngfr in the nucleus but lacked p75Ngfr+ bipolar cytoplasmic extensions. While it is possible that Pax3 expressant cells may associate with glial-derived neurotrophic factor dependent C-fibres (future studies should address this question), the p75Ngfr nuclear expression pattern would be curious; on the other hand, the p75Ngfr/Pax3+ nuclear expression is consistent with that of a peripheral glioblast [40] and is similar to denervated Schwann cell*s in vitro* which when released from axonal contact upregulate p75Ngfr and secrete nerve growth factor for autocrine survival [41].

The morphologic and phenotypic differences of the Pax3 expressing cells described here lend credence to the theory that peripheral glioblasts may be retained in peripheral nerve after birth [42], [43]. Transition from the embryonic Schwann precursor to the ‘committed’, or immature Schwann phenotype progresses at embryonic day 12 in the mouse, at which time changes are associated with the establishment of an autocrine survival circuit. Where precursor Schwann undergo apoptosis in the absence of axonal trophic support, immature Schwann cells survive via autocrine secretion of growth factors such as neurotrophin-3, a ligand of p75Ngfr; at this stage, the fated peripheral glioblasts express *Pax3, p75Ngfr* and *Sox2* and are capable of self-survival [29], [44]–[50]. Thus, it may be theorised that a population of these cells are retained into adulthood and may be the origin of the Pax3/Sox2/p75Ngfr expressant cells described here.

Finally, in the mouse after birth, a subset of Schwann cells that associate with C-fibres differentiate toward a nonmyelinating phenotype, re-establish dependency on paracrine signaling for survival [51] and form the peripheral nonmyelinated nerve bundles. Of note is the finding that 60 day old NMSCs that form the nonmyelinated bundles associated with NGF-dependent C-fibres did not express Pax3, which indicates that expression is down-regulated from postnatal day 30 when Kioussi et al. [14] last report its expression. Thus, Pax3 appears to have a temporal postnatal role in the suppression of myelination genes in a large subset of NMSCs much the same as it does in myelinating Schwann cells [14].

### Prospective Roles for Resident Glioblasts in Adult Peripheral Nerve

Peripheral nerve injuries, in which the nerve trunk is severed, result in separation of the axon from the nucleus and a subsequent inflammatory response called Wallerian degeneration. A fundamental characteristic of Wallerian degeneration is the reported plasticity of adult myelinating Schwann cells to revert from the myelinogenic transcriptional program (or differentiated state) into the cell cycle and back [52]. Briefly, myelinating Schwann cell nuclei enter the DNA synthesis phase while the myelin sheath is relatively intact [53], although re-entry into the cell cycle represents a commitment to demyelination [54]. In the distal stump of the transected nerve, cells discard degraded myelin into cytoplasmic ovoids and initialise autophagocytosis of myelin proteins and lipids [55]–[58] while concomitant inhibition of genes encoding for myelin proteins occurs [59]–[62]. *Pax3* is upregulated at this stage followed by induction of the characteristic immature Schwann cell markers [14]. These immature Schwann cells proliferate within the persisting basal lamina [63], [64] which provides a pathway that proximally regenerating axons use to reach the original target tissue [55], [65]–[68]. As Schwann cells begin to produce and store myelin for remyelination of regenerating axons, *Pax3* levels temporally peak; by contrast, as myelination nears completion, *Pax3* is re-silenced [14]. While theories for the function of Pax3 in myelinated nerve regeneration include prevention of premature myelogenesis and/or orchestration of Schwann progeny migration, development of efficacious methods for identification and visualisation of Pax3 expressing cells in adult peripheral nerve, a primary result of this work, will facilitate future studies of the role of Pax3 in myelinated peripheral nerve regeneration.

Here, the question arises as to why the peripheral nerve trunk would harbour resident glioblasts when myelinating Schwann cells have the capability to de-differentiate. Several suppositions come to mind. Resident glioblasts would proliferate (initially) at a greater rate than a lost myelinating cell, which must extrude and degrade myelin debris before it undergoes proliferation and thus resident glioblasts could be an early source of regenerative Schwann cells. In support of this notion is a study by Griffin et al [69] in which paranodal de-myelination was induced (the myelinated Schwann cells were not lost) and results demonstrated that supernumary Schwann cells had migrated through the endoneurium to overlie areas of paranodal de-myelination. The origin of the supernumary Schwann cells remain unknown, however, the authors suggest that they may have arisen from adjacent nonmyelinated bundles as the supernumary cells appeared situated outside the basal lamina of the damaged myelinated Schwann cells.

Furthermore, there are very few studies outlining the mechanisms of nonmyelinated nerve regeneration and it is possible that resident glioblasts may have a role along these lines. Support for this comes from two studies. Neurofibromatosis Type 1 (NF1) is characterised by loss of the *NF1* gene that encodes neurofibromin [27], [70], [71] and persons affected are predisposed to develop benign peripheral nerve sheath tumours (or neurofibromas), myeloid leukemia, hyperpigmentation of the skin and learning disabilities [72]–[74]; moreover, persons with a loss of heterozygosity of *NF1* alleles develop malignant peripheral nerve sheath tumours [75]. Neurofibromas consist primarily of NMSCs [75]–[77] where malignant transformation is linked to NMSC hyperproliferation and detrimental effects on adjacent cells [78]. Importantly, in neurofibroma, it has been found that tumours have a significant population of stem cells [79] and although the origin of the stem cells remains unknown, Pongpudpunth et al [79] proposed that “formation of neurofibromas may be linked to alterations in the self-renewal program of peripheral nerve progenitor cells”.

Finally, a recent study has demonstrated a population of Pax3+ immature Schwann cells in the cutaneous nerve plexus of the dermis [80]; interestingly, these cells are associated with the unmyelinated nerves of the hair follicle [80], [81]. Based on an understanding of the prominent structural plasticity of the innervation of the hair follicle during hair cycle changes, it is possible that the Pax3+ Schwann cells are resident glioblasts that have a role in the regeneration of unmyelinated nerves during each hair cycle. The Pax3-GFP model holds much potential for the future study of both the Pax3+ Schwann cells described by Djian-Zaouche et al [80] and the further characterisation of the Pax3+ cells described here.

### Conclusion

In 2008, Griffin and Thompson stated that “the possibility of a population of Schwann cell precursors in adult nerves is largely unexplored” [54]. The current investigations were intended to build on the previous work of others who showed that *Pax3*, a classic stem/progenitor cell marker, is detected in adult peripheral nerve trunk. Importantly, our results provide compelling evidence for the existence of glioblasts in adult mouse peripheral nerve and may support the long held theory that embryonic glioblasts are retained in adult peripheral nerve much the same as occurs in other adult Pax3-regulated tissue lineages. Thus, studies are ongoing to further characterise the subset of adult peripheral glial cells described here.

Methods were also developed and described that allowed the visualisation and further characterisation of NMSCs that associate with NGF-dependent C-fibres of normal 60 day old mouse peripheral nerve. To date, neurological studies of this kind have been performed on larger animals such as frog, rat, cat and dog. The intricate and complex morphological characteristics of mouse NMSCs described here, along with the procedures for imaging these cells, provides a foundation for further studies of NMSCs in mouse and may be particularly useful for studies using transgenic animals.

## Materials and Methods

### Animals

Experimental procedures were carried out in accordance with the provisions of the National Health and Medical Research Council Australian Code for Responsible Conduct of Research (2007), the Australian code of practice for the care and use of animals for scientific purposes (2004) and the Animal Welfare Act (2002). Experimentation was approved by the Edith Cowan University Animal Ethics Committee (project approval code 06-A7 ZIMAN). The age of the animals was chosen to reflect the cellular makeup of adult or mature tissue. All the investigations described were undertaken using 60 day old male mice that were provided by the Animal Resources Centre (Canning Vale, Western Australia).

### Isolation of RNA from Sciatic Nerve Specimens

Mice were sacrificed by CO_2_ narcosis at 20%/min v/v and the sciatic nerves were rapidly excised in an aseptic field. Nerves were dissected and ligated under a Leica Zoom 2000 dissecting microscope, with care taken to remove connective fascia from the epineurium. Freshly removed nerve tissue was immediately frozen by immersion in liquid nitrogen and stored at −80°C until further use. Total RNA was isolated from single sciatic nerves using TriReagent (Molecular Research Center, Inc.) and homogenisation with a glass-col mortar and pestle. For each extraction, RNA purity and concentration were assessed using a Bioanalyzer (Agilent).

### RT-PCR

First strand cDNA was synthesised from 2 µg of isolated RNA using an OmniScript system (Qiagen) and an oligo(dT)_18_ primer (10 µM) (Qiagen). Reverse transcription was carried out at 37°C for 1 hour in a total volume of 20 µl. Negative controls included reactions without Omniscript reverse transcriptase. PCR amplifications were performed using a TaqDNA Polymerase Kit (Qiagen) and a negative control (without template DNA) was included in every experiment. The PCR reaction was conducted with the following oligonucleotides, designed using OligoAnalyser 3.1 (Integrated DNA Technologies) and Primer-BLAST (NCBI):

*Pax3c:* (F) 5′-ACCAGGCATGGATTTTCAAG;

(R) 5′-AACGTCCAAGGCTTACTTTG.

*Pax3d:* (F) 5′-CCTCAGGTAATGGGACTTCT;

(R) 5′-AATGAAAGGCACTTTGTCCA.

*Pax3?8:* (F) 5′-CTGTGTCAGATCCCAGCA;

(R) 5′-GAGATAATGAAAGGCACCTGAG.

*Pax3f:* (F) 5′-CAGATGAAGGCTCCGATATTGAC;

(R) 5′-CTGGCTTGAGATAATGAAAGGC.

Internal controls for cDNA were performed using PCR amplification of mouse housekeeping gene *Gapdh* and the primers used were as follows:

*Gapdh:* (F) 5′-GTGAAGGTCGGTGTGAACG;

(R) 5′-ATTTGATGTTAGTGGGGTCTCG.

Positive controls for primers were performed using total RNA isolated from embryonic day 11 mice and PCR negative controls eliminated cDNA as primer template from each PCR reaction. PCR products were resolved on 1.5% w/v agarose gels and visualised under UV light using a Geldoc system. PCR products were sequenced using an ABI PRISM BigDye Terminator Cycle Sequencing Ready Reaction Kit (PE Biosystems) and an ABI Prism 3730 48 capillary sequencer. Sequences were aligned with known sequences in GenBank using the multiAlign tool in Angis, available on GenBank.

### Preparation of Frozen Nerve Sections

To prepare fresh frozen sections of sciatic nerve, animals were sacrificed by cervical disslocation. The sciatic nerves were surgically excised, immersed in Tissue Tek O.C.T. (Sakura Finetek Europe) and frozen in liquid nitrogen cooled N-methyl butane (Sigma). Tissue blocks were cryosectioned at 9 µm onto SuperFrost slides (Menzel-Gläser), dried and fixed in 4% paraformaldehyde in 0.1 M phosphate buffer (PFA) for 30 minutes. Sections were washed in phosphate buffered saline (PBS) 3 times for 5 minutes prior to subsequent processing or storage at −80°C. To prepare pre-fixed frozen sections, animals were anaesthetised with Nembutal (Abbott) and transcardially perfused through the left ventricle; a constant flow (10 mL/min) of PBS (10 mL) followed by ice cold PFA in 0.1 M phosphate buffer at pH 7.4 (50 mL) was established using a peristaltic pump. Sciatic nerves were surgically excised, post-fixed in PFA for 6 hours before immersion in 30% sucrose for 48 hours. Tissues were rinsed in PBS, immersed in Tissue Tek O.C.T. and frozen in liquid nitrogen cooled N-methyl butane prior to cryosectioning at 9 µm onto SuperFrost slides (Menzel-Gläser). Slides were dried prior to processing or storage at −80°C.

### Preparation of Teased Nerve Specimens

To prepare pre-fixed teased nerves, animals were anaesthetised with 75 µg/g Nembutal (Abbott) and transcardially perfused through the left ventricle; a constant flow (10 mL/min) of PBS (10 mL) followed by ice cold paraformaldehyde (PFA) in 0.1 M phosphate buffer at pH 7.4 (50 mL) was established using a peristaltic pump. Sciatic nerves were immediately excised and postfixed in 4% w/v PFA for 18 hours at 4°C. Nerves were rinsed in PBS and prepared onto chilled polylysine slides (Menzel-Gläser) where fascicles were cut into 2 mm lengths and individual fibres were teased apart along the length by 0.2 mm entomology pins. Preparations were dried for 18 hours before immunohistochemical processing or storage at −80°C.

### Preparation of Whole Mount Nerve Fascicle Specimens

Whole mount preparations were prepared using freshly excised nerves which were obtained from animals sacrificed using CO_2_ narcosis. Sciatic nerves were excised and prepared on chilled polylysine slides where fascicles were separated and cut into 2 mm segments and mounted by the epineurium. Slides were dried overnight, post-fixed in acetone for 10 minutes at −20°C and rinsed in PBS at pH 7.4, before immunohistochemical processing or storage at −80°C.

### Antibodies Used for Immunofluorescence

Primary antibodies used were mouse monoclonal IgG2a anti-quail Pax3 (1∶10; Developmental Studies Hybridoma Bank); rabbit polyclonal anti-mouse Krox24 (1∶250; Aviva Systems Biology); rabbit polyclonal anti-mouse Sox2 (1∶200; Sapphire Bioscience) and rabbit polyclonal anti-mouse p75 nerve growth factor receptor (1∶500; Chemicon). Species specific secondary antibodies used were AlexaFluor488-conjugated to goat anti-mouse IgG2a (1∶500; Molecular Probes) and AlexaFluor546-conjugated to goat anti-rabbit IgG (1∶500; Molecular Probes).

### Procedure for Immunofluorescent Staining of Whole Mount Nerve Fascicles

Slides were rehydrated in Tris buffered saline (TBS) and permeabilised in 0.01% v/v Triton X100 (for 45 minutes at 25°C. Slides were washed in TBS 3 times for 10 minutes each prior to incubation in 10% v/v normal goat serum for 6 hours at 25°C. Primary antibodies were individually or simultaneously incubated with 0.2% v/v TritonX100 for 18 hours at 4°C. Specimens were washed in 0.05% v/v TBS/Tween20, 6 times for 30 minutes each, using gentle agitation. Secondary antibody incubation was done thereafter at 25°C for 20 minutes. Specimens were washed in TBS/Tween20, 6 times for 30 minutes using gentle agitation where the last wash contained Hoechst DNA dye 33342 (1 ng/ml). Coverslips were mounted with FluorSave medium (Calbiochem). Negative controls were processed at the same time but were not incubated with primary or secondary antibody.

### Microscopy

Fluorescently labeled tissues were viewed with an Olympus BX51 microscope connected to an Olympus DP71 digital camera and digital images were collected in the Olympus analySIS FIVE program and transferred to the IrfanView (4.27) program for montage construction. The contrast and brightness of these images were unaltered. Whole mount specimens were imaged with a BioRad MRC 1000/1024 UV laser scanning confocal microscope on a Nikon Diaphot 300 with either a 40X objective (with zoom) or 60X immersion objective (without zoom) using a 351- and 488-nanometer argon laser and a 543-nanometer helium/neon laser. Gain and black level adjustments were performed to improve analogue to digital signal conversion and background noise was eliminated using a KALMAN filter. Z-stacks were collected using various step-sizes and KALMAN averaging was performed manually for each step. Digital images were collected and compiled in greyscale and subsequently pseudocoloured with hues approximate to the fluorescence emission spectra of the respective fluorophores using the Confocal Assistant™ (4.02) program. Images were transferred to Adobe Photoshop (7.0) and IrfanView (4.27) programs for montage construction. The images were unaltered.

## References

[1] Epstein DJ, Malo D, Vekemans M, Gros P (1991) Molecular characterization of a deletion encompassing the splotch mutation on mouse chromosome 1. Genomics 10: 89–93. 2045114 10.1016/0888-7543(91)90488-z

[2] Tassabehji M, Read AP, Newton VE, Harris R, Balling R, et al (1992) Waardenburg’s syndrome patients have mutations in the human homologue of the Pax-3 paired box gene. Nature 355: 635–636. 1347148 10.1038/355635a0

[3] Xia SJ, Barr FG (2004) Analysis of the transforming and growth suppressive activities of the PAX3-FKHR oncoprotein. Oncogene 23: 6864–6871. 15286710 10.1038/sj.onc.1207850

[4] Keller C, Arenkiel BR, Coffin CM, El-Bardeesy N, DePinho RA, et al (2004) Alveolar rhabdomyosarcomas in conditional Pax3:Fkhr mice: cooperativity of Ink4a/ARF and Trp53 loss of function. Genes Dev 18: 2614–2626. 15489287 10.1101/gad.1244004 PMC525542

[5] Relaix F, Rocancourt D, Mansouri A, Buckingham M (2005) A Pax3/Pax7-dependent population of skeletal muscle progenitor cells. Nature 435: 948–953. 15843801 10.1038/nature03594

[6] Lang D, Lu MM, Huang L, Engleka KA, Zhang M, et al (2005) Pax3 functions at a nodal point in melanocyte stem cell differentiation. Nature 433: 884–887. 15729346 10.1038/nature03292

[7] Osawa M, Egawa G, Mak SS, Moriyama M, Freter R, et al (2005) Molecular characterization of melanocyte stem cells in their niche. Development 132: 5589–5599. 16314490 10.1242/dev.02161

[8] Relaix F, Montarras D, Zaffran S, Gayraud-Morel B, Rocancourt D, et al (2006) Pax3 and Pax7 have distinct and overlapping functions in adult muscle progenitor cells. J Cell Biol 172: 91–102. 16380438 10.1083/jcb.200508044 PMC2063537

[9] Blake JA, Thomas M, Thompson JA, White R, Ziman M (2008) Perplexing Pax: from puzzle to paradigm. Dev Dyn 237: 2791–2803. 18816860 10.1002/dvdy.21711

[10] Crist CG, Montarras D, Pallafacchina G, Rocancourt D, Cumano A, et al (2009) Muscle stem cell behavior is modified by microRNA-27 regulation of Pax3 expression. Proc Natl Acad Sci U S A 106: 13383–13387. 19666532 10.1073/pnas.0900210106 PMC2726381

[11] Lepper C, Conway SJ, Fan CM (2009) Adult satellite cells and embryonic muscle progenitors have distinct genetic requirements. Nature 460: 627–631. 19554048 10.1038/nature08209 PMC2767162

[12] Medic S, Ziman M (2010) PAX3 expression in normal skin melanocytes and melanocytic lesions (naevi and melanomas). PLoS One 5: e9977. 20421967 10.1371/journal.pone.0009977 PMC2858648

[13] Reeves FC, Burdge GC, Fredericks WJ, Rauscher FJ, Lillycrop KA (1999) Induction of antisense Pax-3 expression leads to the rapid morphological differentiation of neuronal cells and an altered response to the mitogenic growth factor bFGF. J Cell Sci 112 (Pt 2): 253–261. 10.1242/jcs.112.2.253 9858478

[14] Kioussi C, Gross MK, Gruss P (1995) Pax3: a paired domain gene as a regulator in PNS myelination. Neuron 15: 553–562. 7546735 10.1016/0896-6273(95)90144-2

[15] Padilla F, Marc Mege R, Sobel A, Nicolet M (1999) Upregulation and redistribution of cadherins reveal specific glial and muscle cell phenotypes during wallerian degeneration and muscle denervation in the mouse. J Neurosci Res 58: 270–283. 10502283

[16] Gershon TR, Oppenheimer O, Chin SS, Gerald WL (2005) Temporally regulated neural crest transcription factors distinguish neuroectodermal tumors of varying malignancy and differentiation. Neoplasia 7: 575–584. 16036108 10.1593/neo.04637 PMC1501286

[17] Barber TD, Barber MC, Cloutier TE, Friedman TB (1999) PAX3 gene structure, alternative splicing and evolution. Gene 237: 311–319. 10521655 10.1016/s0378-1119(99)00339-x

[18] Pritchard C, Grosveld G, Hollenbach AD (2003) Alternative splicing of Pax3 produces a transcriptionally inactive protein. Gene 305: 61–69. 12594042 10.1016/s0378-1119(02)01186-1

[19] Tsukamoto K, Nakamura Y, Niikawa N (1994) Isolation of two isoforms of the PAX3 gene transcripts and their tissue-specific alternative expression in human adult tissues. Hum Genet 93: 270–274. 7545913 10.1007/BF00212021

[20] Goulding MD, Chalepakis G, Deutsch U, Erselius JR, Gruss P (1991) Pax-3, a novel murine DNA binding protein expressed during early neurogenesis. Embo J 10: 1135–1147. 2022185 10.1002/j.1460-2075.1991.tb08054.x PMC452767

[21] Carlsen F, Behse F (1980) Three dimensional analysis of Schwann cells associated with unmyelinated nerve fibres in human sural nerve. J Anat 130: 545–557. 7410198 PMC1233173

[22] Murinson BB, Hoffman PN, Banihashemi MR, Meyer RA, Griffin JW (2005) C-fiber (Remak) bundles contain both isolectin B4-binding and calcitonin gene-related peptide-positive axons. J Comp Neurol 484: 392–402. 15770655 10.1002/cne.20506

[23] Guenard V, Montag D, Schachner M, Martini R (1996) Onion bulb cells in mice deficient for myelin genes share molecular properties with immature, differentiated non-myelinating, and denervated Schwann cells. Glia 18: 27–38. 8891689 10.1002/(SICI)1098-1136(199609)18:1<27::AID-GLIA3>3.0.CO;2-0

[24] Venters SJ, Argent RE, Deegan FM, Perez-Baron G, Wong TS, et al (2004) Precocious terminal differentiation of premigratory limb muscle precursor cells requires positive signalling. Dev Dyn 229: 591–599. 14991714 10.1002/dvdy.20016

[25] Kury P, Greiner-Petter R, Cornely C, Jurgens T, Muller HW (2002) Mammalian achaete scute homolog 2 is expressed in the adult sciatic nerve and regulates the expression of Krox24, Mob-1, CXCR4, and p57kip2 in Schwann cells. J Neurosci 22: 7586–7595. 12196582 10.1523/JNEUROSCI.22-17-07586.2002 PMC6758000

[26] Topilko P, Levi G, Merlo G, Mantero S, Desmarquet C, et al (1997) Differential regulation of the zinc finger genes Krox-20 and Krox-24 (Egr-1) suggests antagonistic roles in Schwann cells. J Neurosci Res 50: 702–712. 9418958 10.1002/(SICI)1097-4547(19971201)50:5<702::AID-JNR7>3.0.CO;2-L

[27] Le N, Nagarajan R, Wang JY, Araki T, Schmidt RE, et al (2005) Analysis of congenital hypomyelinating Egr2Lo/Lo nerves identifies Sox2 as an inhibitor of Schwann cell differentiation and myelination. Proc Natl Acad Sci U S A 102: 2596–2601. 15695336 10.1073/pnas.0407836102 PMC548989

[28] Topilko P, Schneider-Maunoury S, Levi G, Baron-Van Evercooren A, Chennoufi AB, et al (1994) Krox-20 controls myelination in the peripheral nervous system. Nature 371: 796–799. 7935840 10.1038/371796a0

[29] Murphy P, Topilko P, Schneider-Maunoury S, Seitanidou T, Baron-Van Evercooren A, et al (1996) The regulation of Krox-20 expression reveals important steps in the control of peripheral glial cell development. Development 122: 2847–2857. 8787758 10.1242/dev.122.9.2847

[30] Garratt AN, Voiculescu O, Topilko P, Charnay P, Birchmeier C (2000) A dual role of erbB2 in myelination and in expansion of the schwann cell precursor pool. J Cell Biol 148: 1035–1046. 10704452 10.1083/jcb.148.5.1035 PMC2174554

[31] Gnocchi VF, White RB, Ono Y, Ellis JA, Zammit PS (2009) Further characterisation of the molecular signature of quiescent and activated mouse muscle satellite cells. PLoS One 4: e5205. 19370151 10.1371/journal.pone.0005205 PMC2666265

[32] Adameyko I, Lallemend F, Furlan A, Zinin N, Aranda S, et al (2012) Sox2 and Mitf cross-regulatory interactions consolidate progenitor and melanocyte lineages in the cranial neural crest. Development 139: 397–410. 22186729 10.1242/dev.065581 PMC4067268

[33] Fernandes KJ, Toma JG, Miller FD (2008) Multipotent skin-derived precursors: adult neural crest-related precursors with therapeutic potential. Philos Trans R Soc Lond B Biol Sci 363: 185–198. 17282990 10.1098/rstb.2006.2020 PMC2605494

[34] Kruger GM, Mosher JT, Bixby S, Joseph N, Iwashita T, et al (2002) Neural crest stem cells persist in the adult gut but undergo changes in self-renewal, neuronal subtype potential, and factor responsiveness. Neuron 35: 657–669. 12194866 10.1016/s0896-6273(02)00827-9 PMC2728576

[35] Li L, Fukunaga-Kalabis M, Yu H, Xu X, Kong J, et al (2010) Human dermal stem cells differentiate into functional epidermal melanocytes. J Cell Sci 123: 853–860. 20159965 10.1242/jcs.061598 PMC2831759

[36] Wakamatsu Y, Endo Y, Osumi N, Weston JA (2004) Multiple roles of Sox2, an HMG-box transcription factor in avian neural crest development. Dev Dyn 229: 74–86. 14699579 10.1002/dvdy.10498

[37] Takahashi K, Yamanaka S (2006) Induction of pluripotent stem cells from mouse embryonic and adult fibroblast cultures by defined factors. Cell 126: 663–676. 16904174 10.1016/j.cell.2006.07.024

[38] Masui S, Nakatake Y, Toyooka Y, Shimosato D, Yagi R, et al (2007) Pluripotency governed by Sox2 via regulation of Oct3/4 expression in mouse embryonic stem cells. Nat Cell Biol 9: 625–635. 17515932 10.1038/ncb1589

[39] Ohta S, Imaizumi Y, Okada Y, Akamatsu W, Kuwahara R, et al (2011) Generation of human melanocytes from induced pluripotent stem cells. PLoS One 6: e16182. 21249204 10.1371/journal.pone.0016182 PMC3020956

[40] Wong CE, Paratore C, Dours-Zimmermann MT, Rochat A, Pietri T, et al (2006) Neural crest-derived cells with stem cell features can be traced back to multiple lineages in the adult skin. J Cell Biol 175: 1005–1015. 17158956 10.1083/jcb.200606062 PMC2064709

[41] Sobue G (1990) [The role of Schwann cells in peripheral nerve degeneration and regeneration–NGF-NGF receptor system]. Rinsho Shinkeigaku 30: 1358–1360. 1966016

[42] Davids LM, du Toit E, Kidson SH, Todd G (2009) A rare repigmentation pattern in a vitiligo patient: a clue to an epidermal stem-cell reservoir of melanocytes? Clin Exp Dermatol 34: 246–248. 18828846 10.1111/j.1365-2230.2008.02793.x

[43] Cramer SF (2009) Stem cells for epidermal melanocytes–a challenge for students of dermatopathology. Am J Dermatopathol 31: 331–341. 19461236 10.1097/DAD.0b013e31819cd0cb

[44] Jessen KR, Mirsky R (1992) Schwann cells: early lineage, regulation of proliferation and control of myelin formation. Curr Opin Neurobiol 2: 575–581. 1422113 10.1016/0959-4388(92)90021-c

[45] Jessen KR, Mirsky R (1994) Neural development. Fate diverted. Curr Biol 4, 824–827. 10.1016/s0960-9822(00)00183-4 7820553

[46] Dong Z, Sinanan A, Parkinson D, Parmantier E, Mirsky R, et al (1999) Schwann cell development in embryonic mouse nerves. J Neurosci Res 56: 334–348. 10340742 10.1002/(SICI)1097-4547(19990515)56:4<334::AID-JNR2>3.0.CO;2-#

[47] Grinspan JB, Marchionni MA, Reeves M, Coulaloglou M, Scherer SS (1996) Axonal interactions regulate Schwann cell apoptosis in developing peripheral nerve: neuregulin receptors and the role of neuregulins. J Neurosci 16: 6107–6118. 8815893 10.1523/JNEUROSCI.16-19-06107.1996 PMC6579198

[48] Syroid DE, Maycox PR, Burrola PG, Liu N, Wen D, et al (1996) Cell death in the Schwann cell lineage and its regulation by neuregulin. Proc Natl Acad Sci U S A 93: 9229–9234. 8799183 10.1073/pnas.93.17.9229 PMC38624

[49] Dong Z, Brennan A, Liu N, Yarden Y, Lefkowitz G, et al (1995) Neu differentiation factor is a neuron-glia signal and regulates survival, proliferation, and maturation of rat Schwann cell precursors. Neuron 15: 585–596. 7546738 10.1016/0896-6273(95)90147-7

[50] Meier C, Parmantier E, Brennan A, Mirsky R, Jessen KR (1999) Developing Schwann cells acquire the ability to survive without axons by establishing an autocrine circuit involving insulin-like growth factor, neurotrophin-3, and platelet-derived growth factor-BB. J Neurosci 19: 3847–3859. 10234017 10.1523/JNEUROSCI.19-10-03847.1999 PMC6782711

[51] Chen S, Rio C, Ji RR, Dikkes P, Coggeshall RE, et al (2003) Disruption of ErbB receptor signaling in adult non-myelinating Schwann cells causes progressive sensory loss. Nat Neurosci 6: 1186–1193. 14555954 10.1038/nn1139

[52] Salzer JL, Bunge RP (1980) Studies of Schwann cell proliferation. I. An analysis in tissue culture of proliferation during development, Wallerian degeneration, and direct injury. J Cell Biol 84: 739–752. 6244318 10.1083/jcb.84.3.739 PMC2110577

[53] Stoll G, Griffin JW, Li CY, Trapp BD (1989) Wallerian degeneration in the peripheral nervous system: participation of both Schwann cells and macrophages in myelin degradation. J Neurocytol 18: 671–683. 2614485 10.1007/BF01187086

[54] Griffin JW, Thompson WJ (2008) Biology and pathology of nonmyelinating Schwann cells. Glia 56: 1518–1531. 18803315 10.1002/glia.20778

[55] Weinberg HJ, Spencer PS (1978) The fate of Schwann cells isolated from axonal contact. J Neurocytol 7: 555–569. 722316 10.1007/BF01260889

[56] Perry VH, Brown MC (1992) Role of macrophages in peripheral nerve degeneration and repair. Bioessays 14: 401–406. 1323962 10.1002/bies.950140610

[57] Fernandez-Valle C, Bunge RP, Bunge MB (1995) Schwann cells degrade myelin and proliferate in the absence of macrophages: evidence from in vitro studies of Wallerian degeneration. J Neurocytol 24: 667–679. 7500122 10.1007/BF01179817

[58] Stoll G, Muller HW (1999) Nerve injury, axonal degeneration and neural regeneration: basic insights. Brain Pathol 9: 313–325. 10219748 10.1111/j.1750-3639.1999.tb00229.x PMC8098499

[59] Gupta SK, Poduslo JF, Dunn R, Roder J, Mezei C (1990) Myelin-associated glycoprotein gene expression in the presence and absence of Schwann cell-axonal contact. Dev Neurosci 12: 22–33. 1688760 10.1159/000111832

[60] LeBlanc AC, Poduslo JF (1990) Axonal modulation of myelin gene expression in the peripheral nerve. J Neurosci Res 26: 317–326. 1697906 10.1002/jnr.490260308

[61] Spreyer P, Schaal H, Kuhn G, Rothe T, Unterbeck A, et al (1990) Regeneration-associated high level expression of apolipoprotein D mRNA in endoneurial fibroblasts of peripheral nerve. Embo J 9: 2479–2484. 1695148 10.1002/j.1460-2075.1990.tb07426.x PMC552276

[62] Scherer SS, Xu YT, Bannerman PG, Sherman DL, Brophy PJ (1995) Periaxin expression in myelinating Schwann cells: modulation by axon-glial interactions and polarized localization during development. Development 121: 4265–4273. 8575326 10.1242/dev.121.12.4265

[63] Pellegrino RG, Politis MJ, Ritchie JM, Spencer PS (1986) Events in degenerating cat peripheral nerve: induction of Schwann cell S phase and its relation to nerve fibre degeneration. J Neurocytol 15: 17–28. 3086507 10.1007/BF02057901

[64] Baichwal RR, DeVries GH (1989) A mitogen for Schwann cells is derived from myelin basic protein. Biochem Biophys Res Commun 164: 883–888. 2479378 10.1016/0006-291x(89)91541-6

[65] Ide C, Tohyama K, Yokota R, Nitatori T, Onodera S (1983) Schwann cell basal lamina and nerve regeneration. Brain Res 288: 61–75. 6661636 10.1016/0006-8993(83)90081-1

[66] Salonen V, Peltonen J, Roytta M, Virtanen I (1987) Laminin in traumatized peripheral nerve: basement membrane changes during degeneration and regeneration. J Neurocytol 16: 713–720. 3320279 10.1007/BF01637662

[67] Tona A, Perides G, Rahemtulla F, Dahl D (1993) Extracellular matrix in regenerating rat sciatic nerve: a comparative study on the localization of laminin, hyaluronic acid, and chondroitin sulfate proteoglycans, including versican. J Histochem Cytochem 41: 593–599. 8450198 10.1177/41.4.8450198

[68] Ara J, Bannerman P, Shaheen F, Pleasure DE (2005) Schwann cell-autonomous role of neuropilin-2. J Neurosci Res 79: 468–475. 15635621 10.1002/jnr.20370

[69] Griffin JW, Drucker N, Gold BG, Rosenfeld J, Benzaquen M, et al (1987) Schwann cell proliferation and migration during paranodal demyelination. J Neurosci 7: 682–699. 3559707 10.1523/JNEUROSCI.07-03-00682.1987 PMC6569081

[70] Gutmann DH, Hirbe AC, Haipek CA (2001) Functional analysis of neurofibromatosis 2 (NF2) missense mutations. Hum Mol Genet 10: 1519–1529. 11448944 10.1093/hmg/10.14.1519

[71] Theos A, Korf BR (2006) Pathophysiology of neurofibromatosis type 1. Ann Intern Med 144: 842–849. 16754926 10.7326/0003-4819-144-11-200606060-00010

[72] Cichowski K, Jacks T (2001) NF1 tumor suppressor gene function: narrowing the GAP. Cell 104: 593–604. 11239415 10.1016/s0092-8674(01)00245-8

[73] Riccardi VM (2000) Histogenesis control genes: embryology, wound-healing, and NF1. Teratology 62: 4. 10861625 10.1002/1096-9926(200007)62:1<4::AID-TERA2>3.0.CO;2-Q

[74] Zhu Y, Ghosh P, Charnay P, Burns DK, Parada LF (2002) Neurofibromas in NF1: Schwann cell origin and role of tumor environment. Science 296: 920–922. 11988578 10.1126/science.1068452 PMC3024710

[75] Serra E, Rosenbaum T, Winner U, Aledo R, Ars E, et al (2000) Schwann cells harbor the somatic NF1 mutation in neurofibromas: evidence of two different Schwann cell subpopulations. Hum Mol Genet 9: 3055–3064. 11115850 10.1093/hmg/9.20.3055

[76] Rutkowski JL, Wu K, Gutmann DH, Boyer PJ, Legius E (2000) Genetic and cellular defects contributing to benign tumor formation in neurofibromatosis type 1. Hum Mol Genet 9: 1059–1066. 10767330 10.1093/hmg/9.7.1059

[77] Sheela S, Riccardi VM, Ratner N (1990) Angiogenic and invasive properties of neurofibroma Schwann cells. J Cell Biol 111: 645–653. 1696266 10.1083/jcb.111.2.645 PMC2116200

[78] Zheng H, Chang L, Patel N, Yang J, Lowe L, et al (2008) Induction of abnormal proliferation by nonmyelinating schwann cells triggers neurofibroma formation. Cancer Cell 13: 117–128. 18242512 10.1016/j.ccr.2008.01.002

[79] Pongpudpunth M, Bhawan J, Al-Natour SH, Mahalingam M (2010) Nestin-positive stem cells in neurofibromas from patients with neurofibromatosis type 1-tumorigenic or incidental? Am J Dermatopathol 32: 574–577. 20520523 10.1097/DAD.0b013e3181cc8c7c

[80] Djian-Zaouche J, Campagne C, Reyes-Gomez E, Gadin-Czerw S, Bernex F, et al (2012) Pax3(GFP ), a new reporter for the melanocyte lineage, highlights novel aspects of PAX3 expression in the skin. Pigment Cell Melanoma Res 25: 545–554. 22621661 10.1111/j.1755-148X.2012.01024.x

[81] Botchkarev VA, Eichmuller S, Johansson O, Paus R (1997) Hair cycle-dependent plasticity of skin and hair follicle innervation in normal murine skin. J Comp Neurol 386: 379–395. 9303424 10.1002/(sici)1096-9861(19970929)386:3<379::aid-cne4>3.0.co;2-z

